# Performance and reference intervals of thrombin generation test: results from the Brazilian longitudinal study of adult health (ELSA-Brasil). A cross-sectional study

**DOI:** 10.1590/1516-3180.2021.0425.R1.07102021

**Published:** 2022-04-02

**Authors:** Danyelle Romana Alves Rios, Sandhi Maria Barreto, Letícia Gonçalves Resende Ferreira, Thaís Resende Batista, Ana Paula Ferreira Silva, Wander Valadares de Oliveira, Chams Bicalho Maluf, Maria das Graças Carvalho, Roberta Carvalho Figueiredo

**Affiliations:** I PhD. Associate Professor, Campus Centro Oeste, Universidade Federal de São João del-Rei (UFSJ), Divinópolis (MG), Brazil.; II PhD. Full Professor, Department of Preventive Medicine, Faculdade de Medicina, Universidade Federal de Minas Gerais (UFMG), Belo Horizonte (MG), Brazil.; III MSc. Doctoral Student, Campus Centro Oeste, Universidade Federal de São João del-Rei (UFSJ), Divinópolis (MG), Brazil.; IV MSc. Master’s Student, Campus Centro Oeste, Universidade Federal de São João del-Rei (UFSJ), Divinópolis (MG), Brazil.; V MSc. Master’s Student, Campus Centro Oeste, Universidade Federal de São João del-Rei (UFSJ), Divinópolis (MG), Brazil.; VI MSc. Doctoral Student, Campus Centro Oeste, Universidade Federal de São João del-Rei (UFSJ), Divinópolis (MG), Brazil.; VII PhD. Associate Professor, Department of Clinical Pathology, Faculdade de Medicina, Universidade Federal de Minas Gerais (UFMG), Belo Horizonte (MG), Brazil.; VIII PhD. Full Professor, Campus Centro Oeste, Universidade Federal de São João del-Rei (UFSJ), Divinópolis (MG), Brazil; Full Professor, Department of Clinical and Toxicological Analysis, Faculty of Pharmacy, Universidade Federal de Minas Gerais, Brazil (UFMG), Belo Horizonte (MG), Brazil.; IX PhD. Associate Professor, Campus Centro Oeste, Universidade Federal de São João del-Rei (UFSJ), Divinópolis (MG), Brazil.

**Keywords:** Clinical laboratory techniques, Thrombin, Reference values, Calibrated automated thrombogram, Thrombin generation assay, Reference ranges

## Abstract

**BACKGROUND:**

The thrombin generation test (TGT) has shown promise for investigation of hemorrhagic and thrombotic diseases. However, despite its potential, it still needs standardization. Moreover, few studies have established reference values for TGT parameters. In Brazil, these values have not yet been established.

**OBJECTIVE:**

To determine TGT performance and reference intervals for TGT parameters in healthy individuals.

**DESIGN AND SETTING:**

Cross-sectional study conducted among participants in the Brazilian Longitudinal Study of Adult Health (Estudo Longitudinal de Saúde do Adulto, ELSA-Brasil).

**METHODS:**

The reference sample consisted of 620 healthy individuals. The calibrated automated thrombogram (CAT) method, under low and high tissue factor (TF) conditions, was used to assess thrombin generation. Test performance was analyzed using intra and interassay coefficients of variation (CV) and reference intervals were calculated using the nonparametric method proposed by the International Federation of Clinical Chemistry and the Clinical and Laboratory Standards Institute.

**RESULTS:**

The intraassay CV ranged from 1.4% to 2.2% and the interassay CV, 6.8% to 14.7%. The reference intervals for TGT parameters under low and high TF conditions were, respectively: lagtime: 3.0-10.3 and 1.4-3.7 min; endogenous thrombin potential (ETP): 1134.6-2517.9 and 1413.6-2658.0 nM.min; normalized ETP: 0.6-1.3 and 0.7-1.4; peak: 103.2-397.7 and 256.4-479.0 nM; normalized peak: 0.3-1.3 and 0.7-1.2; and time-to-peak: 5.6-16.0 and 3.4-6.7 min. These parameters were categorized relative to sex.

**Conclusion:**

TGT performance was adequate and the proposed reference intervals were similar to those of other studies. Our findings may be useful for consolidating the TGT, through contributing to its standardization and validation.

## INTRODUCTION

Thrombin has pro and anticoagulant properties and is considered to be the main protein involved in hemostasis regulation.^1^ The thrombin generation test (TGT) is a method that evaluates the capacity of plasma for thrombin generation *ex vivo.*^2^ Unlike the diagnostic and monitoring methods available routinely in laboratories for evaluating hemostasis, in which formation of a fibrin clot occurs when less than 5% of total thrombin has been generated, the TGT is capable of evaluating hemostasis in an overall manner and thus has greater sensitivity.^1,3^ Hence, routine coagulometric methods measure only the initial phase of thrombin generation (TG) and are insensitive to prothrombotic states, while the TGT provides complete information on the phases of propagation and termination. The TGT therefore reflects the components of natural anticoagulation, such as proteins C and S and antithrombin, in addition to the tissue factor pathway inhibitor.^4^

The TGT was developed by Macfarlane and Biggs in the 1950s and later modified by Hemker.^2^ After several further improvements, different methods for measuring thrombin generation were developed.^5,6^

However, the calibrated automated thrombogram (CAT) method is considered to be the reference method.^6^ In this, the proteolytic activity of thrombin generated in plasma is measured using a fluorogenic substrate.^2^ The reaction is triggered through addition of either low or high picomolar concentration of tissue factor (TF), plus phospholipids and calcium ions. Fluorescence is measured continuously for 60 minutes and is proportional to the amount of thrombin generated. The measurements are obtained in a fluorimeter, and the Thrombinoscope software (Thrombinoscope BV, Maastricht, Netherlands) is used to convert the relative fluorescence units (RFU) into thrombin concentration (nM.min), in order to build a thrombin generation curve in real time and calculate its parameters. The main parameters are lagtime (corresponding to the period between addition of trigger reagents and the beginning of thrombin production), peak (maximum thrombin concentration produced in the amplification/propagation phase), time-to-peak (time necessary to reach the maximum thrombin production) and endogenous thrombin potential (ETP) (corresponding to the total amount of thrombin produced, i.e. reflecting the balance between procoagulant and anticoagulant forces).^3^ Extended lagtime and time-to-peak and reduced ETP and peak indicate a state of hypocoagulability. On the other hand, a state of hypercoagulability is characterized by reductions of time-to-peak and lagtime and increased peak and ETP.^7^

From a clinical point of view, a laboratory test that has the capacity to accurately predict the clotting potential of an individual is needed. In this regard, the TGT can be used to understand coagulation mechanisms;^8^ diagnose and monitor hemorrhagic diseases;^9,10^ monitor the use of anticoagulants^4,11^ and antiplatelet agents; and predict recurrence of venous thromboembolism.^12,13,14^

Despite its potential, the TGT still requires standardization and clinical validation studies.^3,6,15^ In fact, some studies have shown that pre-analytical factors can significantly interfere in the TGT and limit its potential for clinical use.^16,17,18,19^ Moreover, only a few studies^20–24^ have determined reference ranges for TGT parameters. ELSA-Brasil was the first major Brazilian study to perform the TGT on a sample of its participants. Establishment of reference values can favor development of clinical studies and implementation of the TGT as a routine laboratory test.

## OBJECTIVE

Therefore, the aim of this study was to evaluate TGT performance and propose reference intervals for TGT parameters, in a sample of healthy participants in the Brazilian Longitudinal Study of Adult Health (Estudo Longitudinal de Saúde do Adulto, ELSA-Brasil). This was done in accordance with the recommendations of the consensus document: “How to define and determine reference intervals in the clinical laboratory”, proposed by the International Federation of Clinical Chemistry and the Clinical and Laboratory Standards Institute (CLSI).^25^

## METHODS

### Population

This study used data from the ELSA-Brasil multicenter cohort study, which involved 15,105 participants (aged 35 to 74 years) who were civil servants working in higher education or research organizations in six Brazilian cities. Detailed information on the baseline of the ELSA-Brasil study has been published elsewhere.^26,27^ Approvals from ethics committees were granted (August 4, 2006; CAAE 0016.1.198.000-06), and all individuals provided their written informed consent.

Out of the 3,115 baseline participants of the state of Minas Gerais, this analysis was restricted to 2,970 individuals from whom TGT were obtained. Additionally, individuals who presented factors that might have affected hemostasis and, therefore, the TGT were excluded, in accordance with the exclusion criteria (Table 1). Out of these 2,970 participants, 2,350 were excluded. Thus, the study population was 620 participants. Moreover, TGT parameters values below the first percentile and/or above the 99^th^ percentile were considered to be outliers and were excluded from the analysis.

**Table 1.** Exclusion criteria among participants and number of excluded individuals

Exclusion criteria	Number of patients (n = 2,970)	
	Excluded (n)	Kept (n)
Continuous use of any medication, including oral contraceptive use and hormone therapy use	1,829	1,141
Self-rating of health as fair, poor or very poor	95	1,046
Self-reported medical diagnosis of diabetes	7	1,039
Self-reported medical diagnosis of arterial hypertension	71	968
Self-reported medical diagnosis of cardiovascular disease^a^	16	952
Self-reported medical diagnosis of thrombosis or pulmonary embolism	19	933
Self-reported medical diagnosis of liver disease^b^	85	848
Self-reported medical diagnosis of cancer	17	831
Current smoker^c^	93	738
Body mass index (BMI) > 30 kg/m^2^	108	630
Glomerular filtration rate (GFR) < 60 ml/min/1.72 m^3^	10	620

^a^Acute myocardial infarction, angina, congestive heart failure, stroke and myocardial revascularization; ^b^cirrhosis or hepatitis; ^c^participants who declared that they had smoked at least one hundred cigarettes over the course of their lives and were still smoking.

### Plasma samples

Venous blood sampling was performed in the mornings after fasting, in accordance with the CLSI Procedures for the Collection of Diagnostic Blood Specimens by Venipuncture: Approved Standard.^28^ Venipuncture was performed for vacuum collection into tubes containing one volume of trisodium citrate (0.105 M) to nine volumes of blood (BD Vacutainer System; Greiner tubes). These were identified using barcodes, to ensure confidentiality, security and traceability of the sample.^29^ Platelet-poor plasma (PPP) was obtained by means of centrifugation at 2,500 g and 4 °C for 15 minutes, and was then stored at -80 °C until use. The time between blood collection and centrifugation was not more than 30 minutes.

### Thrombin generation test

The TGT was performed in PPP using the CAT method (Thrombinoscope BV, Maastricht, Netherlands), using a 96-well plate, under two conditions for triggering the reaction: 1) low TF concentration; and 2) high TF concentration. The CAT method in PPP was carried out as described previously by Duarte et al.^15^ The following TGT parameters were measured and analyzed: lagtime (min), peak (nM), time-to-peak (min) and ETP (nM.min). PPP-reagent low, PPP-reagent high, thrombin calibrator and calcium-containing fluorogenic substrate (FluCa) kit reagents were purchased from Diagnostica Stago (Reading, United Kingdom).

One control plasma pool (CPP) including male participants and another CPP including female participants were prepared in order to normalize the ETP of the other participants’ samples, for internal quality control and determination of intra and interassay variabilities. Each CPP was obtained by mixing 170 samples from female and 170 from male participants in ELSA-Brasil in the state of Minas Gerais, who met the following criteria: C-reactive protein (CRP) ≤ 3 mg/dl (to exclude acute diseases); and not using female hormones or antithrombotic drugs that could potentially interfere with the hemostatic mechanism. The sample aliquots from the participants selected to compose each pool were mixed, aliquoted again and frozen for use during the experiments. CPP was added in duplicate to all plates.

The ETP of the female participants was normalized against the data generated in the female CPP, and the ETP of the male participants was normalized against the data generated in the male CPP, at low and high TF concentrations. According to Dargaud et al.,^16^ normalization of the ETP values of samples, against an ETP value obtained using a CPP, guarantees lower interassay variability in the TGT.

### Statistical analysis

The intra and interassay coefficients of variation (CV) were calculated for all experiments under both conditions (low and high TF), regarding all parameters of the TGT, which was carried out between March and December 2018. CPP was added in duplicate to all plates under the two conditions analyzed. The intraassay variability of the ETP, lagtime, peak and time-to-peak was determined using the CV of the duplicates for each experiment and then averaging all the CVs. The interassay variability of this parameter was determined by calculating the CV between the means of the duplicates of the CPP of all the 164 independent runs that were conducted over the ten-month period.

Skewness and kurtosis tests were applied to evaluate the distribution of TGT parameter values. A nonparametric method was used to determine the reference interval, calculated as the interval from percentile 2.5 to percentile 97.5 of the TGT parameter distribution. Student’s t test was used to verify differences between subgroups defined according to sex and age. We assessed the need to recommend a specific age and sex reference range for the TGT using the Harris/Boyd statistical approach.^25^ Following this approach, we calculated z scores from TGT parameter means and standard deviations (SDs) and compared these with a critical value (z*).

Separate reference intervals are recommended if at least one of the following conditions is met: 1) Calculated z exceeds critical value z*; 2) Statistical differences exist between TGT parameter SDs of each subgroup and the larger SD exceeds the smaller SD 1.5-fold, or if the proportion [larger SD/(larger SD – smaller SD)] is less than 3. It should be mentioned that the CLSI recommends that each subgroup of pre-analytical variables should be composed of at least 120 individuals.^25^

A P-value lower than 0.05 was considered statistically significant, and the analysis was performed using the STATA 9.0 statistical package (Stata; College Station, Texas, United States).

## RESULTS

The population of this study consisted of 620 healthy participants for whom TGT data were available and who were not within the exclusion criteria (Table 1). The majority of the participants were men (56.6%), with ages between 35-54 years (81.6%). They self-declared as white (46.0%) and had reached full higher education (62.4%) (Table 2). In relation to age distribution, there was no statistical difference between men and women (mean age of the men = 47.7 years, SD = 8.0 years; and mean age of the women = 47.2 years, SD = 6.7 years).

**Table 2.** Sociodemographic characteristics of the 620 reference individuals

Characteristics	Frequency	
	n	%
**Sex**		
Male	351	56.6
Female	269	43.4
**Age group (years)**		
35-54	506	81.6
≥ 55	114	18.4
**Self-rated race/skin color**		
White	285	46.0
Brown^a^	231	37.2
Black	75	12.1
Others^b^	29	4.7
**Education (years)**		
< 11	41	6.6
11-14	192	31.0
≥ 15	387	62.4

^a^“Brown” or of mixed color; ^b^includes native indigenous (n = 2), Asian descendent (n = 18) and missing information (n = 9).

The intra- and interassay CVs under low and high TF conditions from the 164 CPP runs are presented in Table 3.

**Table 3.** Intra and interassay coefficients of variation (CVs) of thrombin generation test (TGT) parameters under low and high tissue factor (TF) conditions

TGT parameters		Intraassay CV (%)	Interassay CV (%)
Lagtime	Low TF	1.9	12.4
	High TF	1.7	14.7
ETP	Low TF	1.7	9.3
	High TF	2.2	10.5
Peak	Low TF	2.1	10.4
	High TF	1.7	6.8
Time-to-peak	Low TF	1.9	10.3
	High TF	1.4	10.1

ETP = endogenous thrombin potential.

**Figure 1.**
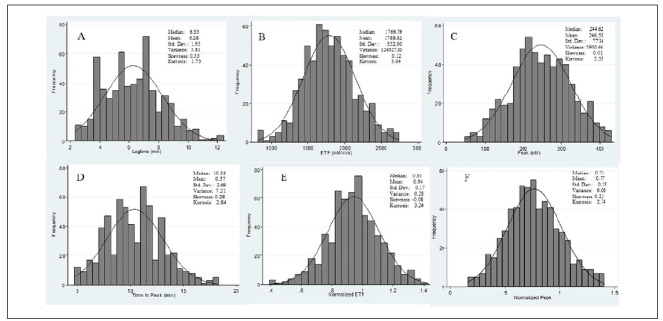
Distribution curves for (A) Lagtime; (B) Endogenous thrombin potential (ETP); (C) Peak; (D) Time-to-peak; (E) Normalized ETP; (F) Normalized peak under the low tissue factor (TF) condition.

**Figure 2.**
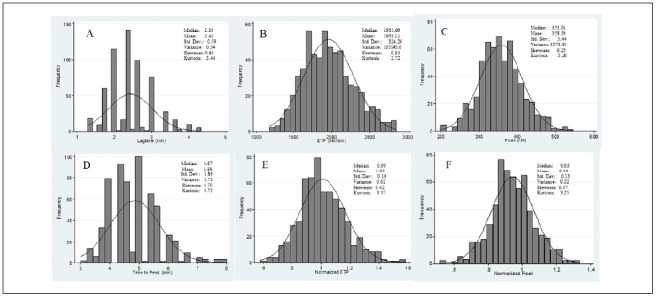
Distribution curves for (A) Lagtime; (B) Endogenous thrombin potential (ETP); (C) Peak; (D) Time-to-peak; (E) Normalized ETP; (F) Normalized peak under the high tissue factor (TF) condition.

Histograms showing the distribution of the TGT parameters are presented in Figures 1 and 2. It can be seen from these that the dispersion of all the parameter values was close to normal distribution.

All TGT parameters were statistically different under both low and high TF conditions and according to sex; and also, for some mean TGT parameters, according to age. Our results relating to sex showed that males had higher mean values for lagtime and time-to-peak and lower mean values for ETP, normalized ETP, peak and normalized peak, in comparison with female participants, under both TF conditions. Regarding age, the lagtime and time-to-peak values under the low TF condition were higher among individuals ≥ 55 years old than among those between 35 and 54 years old. Under the high TF condition, the lagtime and time-to-peak values were higher and peak and normalized peak values were lower among individuals ≥ 55 years old than among those between 35 and 54 years old (Tables 4 and 5).

**Table 4.** Mean, standard deviation and 95% confidence intervals of thrombin generation test (TGT) parameters under the low tissue factor (TF) condition, according to sex and age, among reference individuals (n = 620)

Demographic variable	Parameter	Lagtime (min) (n = 607)	ETP (nM.min) (n = 607)	Peak (nM) (n = 607)	Time to peak (min) (n = 608)	Normalized ETP (n = 597)	Normalized peak (n = 607)
**Sex**							
Male (n = 351)	n Mean (SD) 95% CI	343 6.8 (1.98) 6.5-7.0	345 1724.2 (332.1) 1689.0-1759.3	345 239.3 (72.5) 231.6-246.9	345 10.9 (2.6) 10.6-11.1	333 0.92 (0.16) 0.90-0.93	346 0.72 (0.23) 0.69-0.74
Female (n = 269)	n Mean (SD) 95% CI ^a^P-value	264 5.7 (1.8) 5.5-5.9 0.000	262 1874.0 (361.3) 1830.0-1917.9 0.000	262 256.2 (82.0) 246.3-266.2 0.007	263 9.7 (2.7) 9.4-10.1 0.000	264 0.97 (0.17) 0.95-0.99 0.000	261 0.83 (0.26) 0.80-0.86 0.000
**Age group (years)**							
35 - 54 (n = 506)	n Mean (SD) 95% CI	499 6.1 (2.0) 6.0-6.3	495 1795.5 (356.6) 1764.0-1827.0	496 247.4 (77.2) 240.6-254.2	499 10.2 (2.7) 10.0-10.5	486 0.95 (0.17) 0.93-0.96	495 0.77 (0.25) 0.75-0.79
≥ 55 (n = 114)	n Mean (SD) 95% CI ^a^P-value	108 7.0 (1.7) 6.7-7.3 0.000	112 1759.5 (334.4) 1696.8-1822.1 0.330	111 243.1 (77.3) 228.5-257.6 0.595	109 11.1 (2.5) 10.6-11.5 0.004	111 0.92 (0.16) 0.89-0.95 0.196	112 0.75 (0.25) 0.71-0.80 0.591

The numbers of participants for each parameter differ due to outliers and missing information for each of them (outliers + missing: lagtime male 3 + 5, female 3 + 2; 35-54 years 2 + 5, ≥ 55 years 4 + 2; ETP male 1 + 5, female 5 + 2; 35-54 years 6 + 5, ≥ 55 years 0 + 2; peak male 1 + 5, female 5 + 2; 35-54 years 5 + 5, ≥ 55 years 1 + 2; time to peak male 1 + 5, female 4 + 2; 35-54 years 2 + 5, ≥ 55 years 3 + 2; normalized ETP male 3 + 15, female 3 + 2; 35-54 years 5+15, ≥ 55 years 1 + 2; normalized peak male 0 + 5, female 6 + 2; 35-54 years 6 + 5, ≥ 55 years 0 + 2). ^a^P-value obtained through Student’s t test. ETP = endogenous thrombin potential; SD = standard deviation; CI = confidence interval.

**Table 5.** Mean, standard deviation and 95% confidence intervals of thrombin generation test (TGT) parameters under the high tissue factor (TF) condition, according to sex and age group, among reference individuals

Demographic variable	Parameter	Lagtime (min) (n = 605)	ETP (nM.min) (n = 599)	Peak (nM) (n = 604)	Time to peak (min) (n = 604)	Normalized ETP (n = 593)	Normalized peak (n = 595)
**Sex**							
Male (n = 351)	n Mean (SD) 95% CI	341 2.6 (0.6) 2.5-2.6	341 1905.6 (311.6) 1872.4-1938.8	344 338.6 (47.4) 333.6-343.6	343 5.1 (0.8) 5.0-5.2	333 1.0 (0.2) 0.99-1.02	341 0.92 (0.1) 0.91-0.93
Female (n = 269)	n Mean (SD) 95% CI ^a^P-value	264 2.2 (0.5) 2.2-2.3 0.000	258 2029.8 (327.7) 1989.6-2070.0 0.000	260 384.8 (54.4) 378.2-391.5 0.000	261 4.6 (0.8) 4.5-4.7 0.000	260 1.0 (0.2) 1.01-1.05 0.051	254 0.97 (0.12) 0.96-0.99 0.000
**Age group (years)**							
35-54 (n = 506)	n Mean (SD) 95% CI	493 2.4 (0.6) 2.3-2.4	488 1967.7 (355.7) 1937.8-1997.6	492 362.6 (55.7) 357.7-367.6	492 4.8 (0.8) 4.7-4.8	481 1.0 (0.2) 1.00-1.03	484 0.95 (0.12) 0.94-0.96
≥ 55 (n = 114)	n Mean (SD) 95% CI ^a^P-value	112 2.7 (0.6) 2.6-2.8 0.000	111 1921.4 (266.3) 1871.3-1971.5 0.175	112 340.4 (50.6) 330.9-349.9 0.000	112 5.3 (0.8) 5.1-5.4 0.000	112 1.0 (0.2) 0.97-1.03 0.243	111 0.91 (0.13) 0.88-0.93 0.001

The numbers of participants for each parameter differ due to outliers and missing information for each of them (outliers + missing: lagtime male 5 + 5, female 1 + 4; 35-54 years 6 + 7, ≥ 55 years 0 + 2; ETP male 5 + 5, female 7 + 4; 35-54 years 11 + 7, ≥ 55 years 1 + 2; peak male 2 + 5, female 4 + 5; 35-54 years 6 + 8, ≥ 55 years 0 + 2; time to peak male 2 + 6, female 3 + 5; 35-54 years 5 + 9, ≥ 55 0 + 2; normalized ETP male 3 + 15, female 2 + 7; 35-54 years 5 + 20, ≥ 55 years 0 + 2; normalized peak male 0 + 8, female 6 + 5; 35-54 years 6 + 11, ≥ 55 years 0 + 2). ^a^P-value obtained through Student’s t test. ETP = endogenous thrombin potential; SD = standard deviation; CI = confidence interval.

Regarding sex, the calculated z for lagtime, ETP and time-to-peak under the low TF condition, and for lagtime, peak, normalized peak and time-to-peak under the high TF condition, was higher than z*. In addition, each category (male and female) had more than 120 participants. Regarding age, the calculated z for lagtime and time-to-peak under the high TF condition was higher than z*, but one category (age ≥ 55 years) had fewer than 120 individuals (n = 114), i.e. it did not meet the CLSI recommendations. Thus, we chose to present reference intervals categorized according to sex only for the parameters that met at least one criterion of the Harris/Boyd statistical approach and the CLSI recommendations.

Table 6 shows the reference intervals for TGT parameters under both low and high TF conditions. As expected, under the low TF condition, ETP and peak values were slightly lower than those obtained under the high TF condition. However, for the lagtime and time-to-peak parameters, the inverse was observed, i.e. higher values for low TF and lower values for high TF.

**Table 6.** Reference intervals for the thrombin generation test (TGT) parameters under low and high tissue factor (TF) conditions

TGT parameters		Median	Reference interval	
			Percentile 2.5	Percentile 97.5
**Low TF condition**				
Lagtime (min)	All	6.3	3.0	10.3
	Male	6.7	3.3	10.7
	Female	5.7	2.7	9.7
ETP (nM.min)	All	1769.8	1134.6	2517.9
	Male	1711.2	1063.0	2393.2
	Female	1860.4	1202.8	2619.0
Peak (nM)	All	244.6	103.2	397.7
Time-to-peak (min)	All	10.3	5.6	16.0
	Male	10.9	6.3	16.1
	Female	9.33	4.7	15.7
Normalized ETP	All	0.9	0.6	1.3
Normalized peak	All	0.7	0.3	1.3
	Male	0.7	0.3	1.2
	Female	0.8	0.4	1.3
**High TF condition**				
Lagtime (min)	All	2.3	1.4	3.7
	Male	2.7	1.6	4.0
	Female	2.3	1.3	3.4
ETP (nM.min)	All	1931.7	1413.6	2658.0
Peak (nM)	All	355.1	256.4	479.0
	Male	335.8	248.9	437.6
	Female	381.8	279.7	499.6
Time-to-peak (min)	All	4.7	3.4	6.7
	Male	5.0	3.6	7.0
	Female	4.3	3.4	6.3
Normalized ETP	All	1.0	0.7	1.4
Normalized peak	All	0.9	0.7	1.2

ETP = endogenous thrombin potential; min = minutes.

## DISCUSSION

Our study, developed using a sample of healthy participants from a large cohort of Brazilian adults, showed adequate TGT performance, as measured through intra- and intertest variability. Furthermore, this study established reference intervals for TGT parameters, and showed that for some parameters, these intervals need to be stratified according to sex.

Use of a standardized procedure for the TGT resulted in acceptable validation criteria, with CVs for most TGT parameters of < 10%.^30^ In our study, the intra and interassay variability of the ETP, lagtime, peak and time-to-peak ranged from 1.4% to 2.2% and from 6.8% to 14.7%, respectively. These findings are in agreement with those of Duarte et al.,^15^ in which the intra-assay CV for all parameters was below 10%; and with those of Ten Cate-Hoek et al.,^23^ in which none of the TGT parameters, under either condition, presented CV greater than 5%. In a study by Bloemen et al.,^20^ the CVs of all parameters were shown to range from 10% to 27%. One explanation for this higher CV could particularly be that individualized reagents of different origins may have been used, which would involve more steps in carrying out the technique; whereas in our study and in the others mentioned, single-manufacturer kits were used.

There is evidence that the TGT is a more sensitive method for assessing hemostasis than routine coagulometric assays, such as prothrombin time, activated partial thromboplastin time or individual coagulation factor assays.^31,32^ The TGT has basically been performed under two experimental conditions: low and high concentrations of TF. Bagot et al.^33^ reported that most researchers now consider that use of a low TF concentration provides greater sensitivity due to its higher capacity for evaluating the intrinsic pathway, natural coagulation inhibitors and fibrinogen. However, the TGT using high TF concentration may be useful for evaluating more hypercoagulable states, i.e. when patients are using anticoagulants,^6^ and for analyses to investigate the natural anticoagulation mechanism, through addition of activated protein C^34^ or thrombomodulin.^35,36,37^

In accordance with most of the studies summarized in Table 7, we also proposed and performed determination of TGT reference intervals under both experimental conditions, with low and high TF, and our findings were similar to those of these previous studies. Only Lundbech et al.^21^ and Haidl et al.^24^ conducted evaluations only with low or high TF, respectively. The five studies presented were carried out in Austria, Germany, Denmark and Holland, and used the CAT method to evaluate thrombin generation. In four of them, Diagnostica Stago or Thrombinoscope BV kits were acquired. Only one study, Bloemen et al.,^20^ used individualized reagents from different sources. The studies by van Paridon et al.^22^ and Lundbech et al.^21^ stratified the reference intervals according to sex, while Bloemen et al.,^20^ Ten Cate-Hoek et al.^23^ and Haidl et al.^24^ did not stratify.

**Table 7.** Reference intervals for thrombin generation test (TGT) parameters from different studies

Study	Subject population	Country of origin	Method/equipment/reagents	Reference intervals to PPP	
				Low condition (TF ∼1 pM)	High condition (TF ∼5 pM)
ELSA-Brasil	620 healthy adult individuals aged 35-74 years	Brazil	– CAT/ Fluoroskan Ascent^TM^ microplate fluorometer – Kits were obtained from Diagnostica Stago	2.5%-97.5% • Lagtime (min): 3.0-10.3 Male: 3.3-10.7 Female: 2.7-9.7 • ETP (nM.min): 1134.6-2517.9 Male: 1063.0-2393.2 Female: 1202.8-2619.0 • Peak (nM): 103.2-397.7 • Time-to-peak (min): 5.6-16.0 Male: 6.3-16.1 Female: 4.7-15.7 • Normalized ETP: 0.9-1.3	2.5%-97.5% • Lagtime (min): 1.4-3.7 Male: 1.6-4.0 Female: 1.3-3.4 • ETP (nM.min): 1413.6-2658.0 • Peak (nM): 256.4-479.0 Male: 248.9-437.6 Female: 276.7-499.6 • Time-to-peak (min): 3.4-6.7 Male: 3.6-7.0 Female: 3.4-6.3 • Normalized ETP: 0.7-1.4
Lundbech et al., 2020^21^	124 blood donors aged 21-66 years	Denmark	– CAT/ Fluoroskan Ascent^TM^ microplate fluorometer – Kits were obtained from Thrombinoscope BV	X ± 1.96*SD Subgroups: 95% CI • Lagtime (min): 4.4-9.4 Male: 7.1-7.6 Female: 6.4-6.7 • ETP (nM.min): 554.0-1952.0 Male: 1093.0-1258.0 Female: 1249.0-1440.0 • Peak (nM): 46.0-288.0 Male: 139.0-164.0 Female: 167.0-203.0 • Time-to-peak (min): 8.0-15.0 Male: 11.7-12.5 Female: 10.3-11.2	NA
Van Paridon et al., 2019^22^	1,210 apparently cardiovascularly healthy subjects without history of CVD (myocardial infarction, congestive heart failure, coronary artery disease, venous thromboembolism, atrial fibrillation or peripheral artery disease), presence of CVRFs (obesity, dyslipidemia, arterial hypertension or diabetes mellitus) or use of antithrombotic agents, oral contraceptives or hormonal replacement therapy. The median age was 47 years (IQR 42-55) among males and 48 years (IQR 45-55) among females.	Germany	– CAT/ Fluoroskan Ascent^TM^ microplate fluorometer – Kits were obtained from Thrombinoscope BV	Lagtime : median (IQR) ETP and Peak: mean (SD) • Lagtime (min): Male: 5.07 (4.67-5.67) Female: 4.67 (4.33-5.33) • ETP (nM.min): Male: 1047 (216) Female: 1099 (203) • Peak (nM): Male: 108 (51) Female: 115 (48.7) • Time-to-peak (min): NA	Lagtime : median (IQR) ETP and Peak: mean (SD) • Lagtime (min): Male: 2.67 (2.33-3.00) Female: 2.39 (2.33-2.67) • ETP (nM.min): Male: 1322 (196) Female: 1318 (212) • Peak (nM): Male: 236 (52.2) Female: 259 (53.3) • Time-to-peak (min): NA
Bloemen et al., 2017^20^	129 healthy adult individuals (did not have any predisposition to/history of thrombosis or bleeding or they had not taken any oral anticoagulant or antiplatelet drugs for at least 2 weeks before testing). The median age was 32.0 years (IQR 27.0-43.5).	Netherlands	– CAT/ Fluoroskan Ascent^TM^ microplate fluorometer – Individualized reagents from different sources	2.5%-97.5% • Lagtime (min): 3.3-5.8 • ETP (%): 72.3-141.5 • Peak (%): 30.5-97.2 • Time-to-peak (min): 6.1-10.9	2.5%-97.5% • Lagtime (min): 1.7-2.9 • ETP (%): 77.7-142.9 • Peak (%): 73.3-126.9 • Time-to-peak (min): 3.1-5.0
Ten Cate-Hoek et al., 2008^23^	137 healthy individuals (without anticoagulant, anti-platelet or oral contraceptives drugs, and no pregnant women) recruited from the community. Mean age was 53.7 years.	Netherlands	– CAT/ Fluoroskan Ascent^TM^ microplate fluorometer – Kits were obtained from Thrombinoscope BV	95% CI • Lagtime (min): 3.9-4.1 • ETP (%): 99.0-106.3 • Peak (%): 92.7-105.5	95% CI • Lagtime (min): 2.4-2.8 • ETP (%): 96.6-109.2 • Peak (%): 89.7-97.5
Haidl et al., 2006^24^	35 healthy adult volunteers consisting of students and medical staff who were not taking any medication that would influence coagulation aged < 35 years.	Austria	– CAT/ Fluoroskan Ascent^TM^ microplate 9 fluorometer – Individualized reagents from different sources	NA	Mean ± 2-fold SD • Lagtime (min): 1.35-2.39 • ETP (nM.min): 1745.0-2737.0 • Peak (nM): 433.0-669.0 • Time-to-peak (min): 2.75-4.31

TF = tissue factor; ETP = endogenous thrombin potential; CAT= calibrated automated thrombogram; CVD = cardiovascular disease; CVRFs = cardiovascular risk factors; SD = standard deviation; CI = confidence interval; PPP = platelet-poor plasma; ELSA = Longitudinal Study of Adult Health (Estudo Longitudinal de Saúde do Adulto); IQR = interquartile range; NA = not applicable; min = minutes.

In our study, significant differences according to sex were found in the means of all TGT parameters, under both conditions. Women had lower lagtime and time-to-peak averages, and higher ETP, peak and normalized peak, i.e. they had higher thrombin generation than men. These findings are similar to those of the study by van Paridon et al.,^22^ who suggested that female endogenous sex hormones had an influence on hemostasis, such that this would increase the fibrinogen levels and reduce the natural anticoagulant levels. This would explain the differences in TGT parameter values between the sexes.^38^

Regarding age, the mean lagtime and time-to-peak under low TF and high TF conditions were higher and the mean peak and normalized peak were lower only with high TF, among individuals aged 55 years or over, in comparison with those aged between 35 and 54 years. Some other studies have described the effect of age on the TGT.^22,24,39,40^ Van Paridon et al.^22^ observed that there was a positive association between lagtime and age among both men and women, which corroborates our findings. They also observed that ETP and the peak increased with age among both men and women, and that the potential for thrombin generation tended to decrease with age. Other studies have generally suggested that a positive association exists between thrombin generation and increasing age.^24,39,40^ It should be noted that these studies had small samples. We therefore believe that both the previous findings and our findings relating to age still need to be better clarified.

It is known that the plasma levels of hemostatic factors can present racial differences. In general, blacks have higher levels of factors VII and VIII and lower levels of proteins C and S than those of whites.^41,42,43,44^ However, the data on possible racial differences relating to the potential for thrombin generation in a healthy population remain inconclusive.^45^ In our study, no significant differences in TGT parameters were observed in relation to the race/color of the participants (data not presented). However, it should be noted that color/race was obtained through self-reporting. Pena et al.^46^ showed that in Brazil, skin color, phenotypically evaluated, has a very weak correlation with the degree of ancestry.

The reference ranges in this study were determined in accordance with the CLSI recommendations. We presented general and sex-stratified reference intervals for the parameters of lagtime, ETP, time-to-peak and normalized peak under the low TF condition and lagtime, peak and time-to-peak under the high TF condition. For these parameters, the results from the Harris and Boyd test showed that the calculated z of these parameters exceeded the z*, thus indicating that reference intervals categorized according to sex would be clinically useful.^25^ However, we did not present reference intervals according to age category, because only two parameters (lagtime and time-to-peak under the high TF condition) showed calculated z greater than z*. Moreover, one category (age ≥ 55 years) had fewer than 120 individuals (n = 114) and thus did not meet the CLSI recommendations.

One of the greatest challenges in studies in which the proposal is to determine reference intervals for biological analytes is how to select of healthy individuals. Most studies use convenience sampling, consisting of medical students, blood donors, etc. This is questionable because these groups are not representative of the population in which these parameters are evaluated.^25,47,48^ Nonetheless, most of the studies that have put forward reference ranges for TGT parameters used samples that were precisely from blood donors,^21^ students and medical staff^24^ or young and healthy adult individuals,^20^ as can be observed in Table 7.

Thus, our study innovated and aggregated information on this topic, since the completeness and quality of the ELSA-Brasil data ensured that a healthy reference sample was selected. This study was developed using a sample from the general population that did not include any individuals with medical diagnoses of diabetes, hypertension, CVD, venous thromboembolism, cancer or liver diseases, or any obese individuals, smokers, individuals with altered glomerular filtration rate (GFR) and individuals making regular use of medications (including female hormones). Hence, conditions that may affect hemostasis were excluded.

Although our reference ranges can be generalized to other populations in order to identify patients with risks of bleeding and thrombosis, it is important to emphasize that these ranges should be used with caution. These values may differ according to the ethnic origin of the population, geographical location or living habits, among other factors.^25^ In addition, pre-analytical variables, including individual preparation; devices and collection tubes (with or without corn trypsin inhibitor, CTI); sample storage time and processing; and the analysis method itself, such as the use of different sources, batches and concentrations of TF and different mixtures of phospholipids (PL), along with proper interpretation of results, may also have an impact on laboratory test values.^16,20,21^ Thus, we emphasize that each laboratory should validate these reference ranges according to the protocols, reagents and equipment used. It should also be noted that internal calibration is necessary to correct intraassay variation, while normalization of ETP and peak against a normal control plasma, which was performed in our study, is important for correcting significant temporal variations and for comparing plasma populations within and between institutions.^40^

One limitation of our study was that the TGT was done on PPP that had been prepared by means of centrifugation on whole blood collected in citrate, in a single stage (15 minutes at 2,500 g). This contrasts with the recommendations, i.e. centrifugation in two stages: 2000 g for 5 minutes and 10,000 g for 10 minutes. Loeffen et al.^30^ showed that double centrifugation is more appropriate for eliminating the interference of platelets and microparticles, which may contribute to the variability in the results from the TGT. Therefore, it cannot be ruled out that this interference could have occurred in our TGT evaluations. Nonetheless, it is worthwhile quoting the words of Tripodi,^49^ regarding the PPP to be tested using the TGT: “Double centrifugation has been advocated, but cannot be used on a regular basis, as it is not the standard practice in general laboratories that work by automated procedures. Furthermore, many samples prepared for general purpose via the standard centrifugation cannot be later used for TGT. An acceptable compromise would be the blood centrifugation at 3,000 g for 15 minutes (controlled room temperature). This procedure would allow getting plasma with minimal residual platelets”. On the basis of Tripodi’s statement, and given that our samples were centrifuged at 2,500 g for 15 minutes, it is likely that they did not contain many residual platelet fragments. Another limitation is that the intraassay CV was derived from only one duplicate per plate, although the most appropriate method would have been use of triplicates or greater numbers of replicates.

On the other hand, this study presents the strong point of being the first to perform the TGT on a large cohort of Brazilian adults, with selection of a good-quality reference sample. It represents additional progress towards standardization and validation of the TGT via the CAT method. Furthermore, a few laboratories in Brazil have worked with and validated protocols for this technique, using internal controls and standardization of parameters, especially ETP.

The implication for practice from our findings is that they may motivate other similar studies in other parts of the world aimed at investigating potential interference in the TGT from pre-analytical factors and consolidating the TGT reference intervals. Such studies will contribute towards standardization and validation of the TGT and, therefore, facilitate its clinical use.

Lastly, it should be noted that our measurement of thrombin generation among the baseline sample of ELSA-Brasil may stimulate studies with the aim of assessing whether TGT parameters, especially ETP, are associated with clinical conditions, especially chronic non-communicable diseases in the Brazilian population, or whether the TGT can be a predictor of mortality when the thrombin level is above the maximum value of the reference interval.

## CONCLUSION

TGT performance was adequate and the proposed reference intervals were similar to those of other studies. Our findings may be useful for consolidating the TGT, through contributing to its standardization and validation.
